# A cytokine mixture of GM-CSF and IL-3 that induces a neuroprotective phenotype of microglia leading to amelioration of (6-OHDA)-induced Parkinsonism of rats

**DOI:** 10.1002/brb3.11

**Published:** 2011-09

**Authors:** Mohammed Emamussalehin Choudhury, Kana Sugimoto, Madoka Kubo, Masahiro Nagai, Masahiro Nomoto, Hisaaki Takahashi, Hajime Yano, Junya Tanaka

**Affiliations:** 1 Department of Therapeutic Medicine, Graduate School of Medicine, Ehime UniversityToon, Ehime, Japan; 2 Department of Molecular and Cellular Physiology, Graduate School of Medicine, Ehime UniversityToon, Ehime, Japan; 3 Department of Basic and Clinical Neuroscience, Ehime Proteo-Medicine Research Center, Ehime UniversityToon, Ehime, Japan

**Keywords:** Astrocyte, dopamine, HGF, 6-hydroxydopamine, neuroinflammation, NG2 glia

## Abstract

Dopamine (DA) agonists are widely used as primary treatments for Parkinson's disease. However, they do not prevent progressive degeneration of dopaminergic neurons, the central pathology of the disease. In this study, we found that subcutaneous injection of a cytokine mixture containing granulocyte macrophage colony-stimulating factor and interleukin-3 (IL-3) markedly suppressed dopaminergic neurodegeneration in 6-hydroxydopamine-lesioned rats, an animal model of Parkinson's disease. The cytokine mixture suppressed the decrease of DA content in the striatum, and ameliorated motor function in the lesioned rats. In response to the cytokine injection, dopaminergic neurons in the substantia nigra pars compacta increased expression of the antiapoptotic protein Bcl-xL. Microglial activation in the pars compacta was evident in both the saline- and cytokine-injected rats. However, the cytokine mixture suppressed expression of the proinflammatory cytokines IL-1β and tumor necrosis factors α, and upregulated the neuroprotective factors insulin-like growth factor-1 and hepatocyte growth factor. Similar responses were observed in cultured microglia. Detailed morphometric analyses revealed that NG2 proteoglycan-expressing glial cells increased in the cytokine-injected rats, while astrocytic activation with increased expression of antioxidative factors was evident only in the saline-injected rats. Thus, the present findings show that the cytokine mixture was markedly effective in suppressing neurodegeneration. Its neuroprotective effects may be mediated by increased expression of Bcl-xL in dopaminergic neurons, and the activation of beneficial actions of microglia that promote neuronal survival. Furthermore, this cytokine mixture may have indirect actions on NG2 proteoglycan-expressing glia, whose role may be implicated in neuronal survival.

## Introduction

Parkinson's disease (PD) is a very common neurodegenerative disorder, which is characterized by resting tremor, impaired balance and coordination, bradykinesia, and rigidity. The main pathology of PD is progressive degeneration of dopaminergic (DArgic) neurons in the substantia nigra, pars compacta (SNpc). DArgic neurodegeneration results in decreased dopamine (DA) content in the striatum, which is the major cause of motor disability in PD. Therefore, current PD treatments are mostly focused on replenishing DArgic activity in the striatum by administering L-DOPA or other DA agonists to PD patients. However, this type of therapy does not suppress the DArgic neurodegeneration. Therefore, novel treatments are being sought that mitigate neuronal loss in PD (Yacoubian and Standaert 2009).

In addition to neurodegeneration, glial cell activation has been shown as a pathologic feature of PD (Mosley et al. 2006; McGeer and McGeer 2008; Tansey and Goldberg 2010). Therefore, it is speculated that treatments that affect glial function in the SNpc can lead to novel PD treatments. There are four types of glial cells in the SNpc: astrocytes, microglia, oligodendrocytes, and NG2 glia. The NG2 glia are glial cells specifically expressing NG2 chondroitin sulphate proteoglycan, and are, at least in part, precursor cells for oligodendrocytes. NG2 glia are sometimes also called NG2 cells, synantocytes, and polydendrocytes (Butt et al. 2005; Staugaitis and Trapp 2009; Trotter et al. 2010).

In response to neuronal injuries, astrocytes become activated, increase their expression of glial fibrillary acidic protein (GFAP), and migrate to the sites of injury. At this point, they are called reactive astrocytes, and form astroglial scars in and around the injury sites. In PD, the accumulation of reactive astrocytes in the SNpc is well documented (McGeer and McGeer 2008; Asanuma et al. 2010; Choudhury et al. 2011). Although the actions of reactive astrocytes on neuronal survival have not yet been fully elucidated, it is believed that they possess neuroprotective attributes, which have mainly been observed in *in vitro* experiments (Tanaka et al. 1999; Miyazaki et al. 2011). The neuroprotective actions of astrocytes have been attributed to their antioxidant defense mechanisms, and their ability to secrete a variety of neuroprotective factors, such as glial cell-line-derived neurotrophic factor (GDNF) (Schaar et al. 1993) and brain-derived neurotrophic factor (BDNF) (Dreyfus et al. 1999). Because of the presumed neuroprotective role of astrocytes, agents targeting these cells have been proposed to suppress DArgic neurodegeneration (Asanuma et al. 2010; Choudhury et al. 2011).

Microglia play pivotal roles in immune reactions in the brain. Microglial cells are mesodermal in origin with macrophage-like properties (Kreutzberg 1996). PD has some features in common with neuroinflammatory diseases, because it is characterized by the presence of activated microglia in the SNpc (Mosley et al. 2006; McGeer and McGeer 2008; Long–Smith et al. 2009; Tansey and Goldberg 2010). Similar neuroinflammatory reactions may be critical in another major neurodegenerative disorder Alzheimer's disease (Lue et al. 2010) and therefore microglia should be the therapeutic targets to suppress neurodegeneration (Yacoubian and Standaert 2009). However, in contrast to astrocytes, the majority appears to support the notion that microglia are detrimental to the disease (Liberatore et al. 1999; Block et al. 2007; Henry et al. 2009; Marinova–Mutafchieva et al. 2009), as they are known to produce proinflammatory cytokines, such as interleukin-1β (IL-1β) and tumor necrosis factors α (TNFα) (Long–Smith et al. 2009; De Lella Ezcurra et al. 2010), and increase oxidative stress (Liberatore et al. 1999; Levesque et al. 2010). Thus, astrocytes and microglia have often been implicated in the pathogenesis of PD. On the other hand, NG2 glia and oligodendrocytes have also been shown to abundantly exist in the SNpc, whereas very little is known about their roles in PD (McGeer and McGeer 2008).

A cytokine mixture of granulocyte macrophage colony-stimulating factor (GM-CSF) and IL-3 has been found of its much stronger ameliorative effect on the stab-wounded rat brains than the solely used GM-CSF or IL-3 (Nishihara et al. 2011). In the present study, the cytokine mixture effectively prevented 6-hydroxydopmaine (6-OHDA)-induced neurodegeneration in the SNpc, which is an animal model of Parkinsonism. The findings suggest that the effects are mediated by increased expression of prosurvival proteins, and the differential activities of neuroinflammatory cells, including NG2 glia, whose role may be implicated in neuronal survival.

## Materials and Methods

### Animals

Adult male Wistar rats, weighing 220–250 g, were housed under standard laboratory conditions. The animals were allowed free access to food and water throughout the experiments. The rats were kept in a 12/12 h dark/light cycle. All animal experiments were carried out in accordance with the Guidelines for Animal Experimentation of Ehime University Graduate School of Medicine.

### 6-OHDA treatment and cytokine injection

Animals were kept under pentobarbital sodium anesthesia (50 mg/kg) and placed in a stereotactic instrument (Narishige, Tokyo, Japan). 6-OHDA (Sigma, St. Louis, MO) was dissolved in saline containing ascorbic acid (Wako, Osaka, Japan) (10 μg/μL dissolved in 1% ascorbate-saline), kept on ice (4°C) and protected from light to minimize oxidation, until use. The rats were then given uni- or bilateral injections of 6-OHDA. Unilateral injection was employed for immunohistochemical analyses, and bilateral injection was used for all other studies. For unilateral injection, 5 μL of 6-OHDA was drawn into a Hamilton syringe and then injected into the right side of the striatum, through a hole made on the skull at 1 mm anterior to bregma and 3 mm lateral from the midline. The depth of the needle tip was 5 mm from the skull surface. The same amount of 6-OHDA was injected into the left side of the striatum for bilateral injection. The rate of fluid injection was 1 μL/min. The needle was left at the point of injection for an additional 10 min after the injection and then slowly withdrawn. Because the bilaterally injected rats could not move well to drink or to eat, they were intraperitoneally injected with electrolyte solution (Solita-T3, Ajinomoto, Tokyo, Japan) twice per day for 1 week. A cytokine mixture containing 0.2 mg/mL rat recombinant GM-CSF (PeproTech, London, UK) and 0.2 mg/mL rat recombinant IL-3 (PeproTech) was subcutaneously injected from the next day of the 6-OHDA-treatment at a dose of 10 μg/kg body weight (Nishihara et al. 2011). For the control, the same amount of saline was subcutaneously injected.

### Determination of DA content in the striatum

The DA content in the striatum was measured by high-performance liquid chromatography (HPLC) (Yabe et al. 2009). Both sides of the striatum were dissected out and quickly put on an ice-cold glass plate and stored at −80°C until assayed. The striatum samples from both sides were independently homogenized with an ultrasonic cell disruptor (Tomy Seiko, Tokyo, Japan) in 0.1 M perchloric acid containing 5 mM EDTA (Wako) and 3,4-dihydroxybenzamine (Wako), and were centrifuged. A 10-μL aliquot of the filtered supernatant was injected into a HPLC apparatus with a reversed-phase column. The mobile phase consisted of 15% (v/v) methanol containing 0.1 M sodium acetate (Wako) and 0.1 M citric acid (Wako), adjusted to pH 3.5, with 180 mg/L sodium octydyl sulphate (Wako), and 10 mM EDTA, pumped through the column at a rate of 0.25 mL/min. The data from the right and left striatum were averaged and processed for statistical analysis.

### Rota-rod test

Motor coordination and balance were tested using a rota-rod (Ugo Basile, Rota-rod 7750, Italy) before administration of drugs, and 7, 14, 21, 28, and 56 days after administration of the drugs. The rota-rod test was performed by placing the rat on a rotating drum and measuring the time each animal was able to maintain its balance while attempting to walk on top of the rod (Dekundy et al. 2007). The test was done between 1400 h and 1500 h. Animals were pretrained twice a day, 3 days before the test. The speed of the rota-rod was maintained fixed at 40 rpm over a 300-s period. The animals were touched on their tails several times in each session to maintain a high degree of alertness in the test. The rota-rod performance was expressed in seconds; namely the amount of time the animals remained on the rotating rod.

### Quantitative real-time reverse transcriptase-polymerase chain reaction (qRT-PCR)

The right side ventral midbrain containing the substantia nigra (midbrain delineated longitudinally 4.5 to 6.6 mm from bregma, perpendicularly under 7 mm from the skull) was dissected out at 7 days after 6-OHDA-treatment and stored at −80°C until assayed. Tissue samples were homogenized in ISOGEN (Nippon gene, Tokyo, Japan) using an ultrasonic cell disruptor. Then, their total RNA was collected. cDNA was obtained from DNase-I-treated RNA by reverse transcription using an oligo-(dT) 15 primer, as previously described (Takahashi et al. 2008). cDNA samples were prepared from seven separate samples of brain tissue astrocytes and microglia. Quantitative real-time reverse transcriptase PCR (qRT-PCR) analysis was performed in triplicate using a MJ mini instrument (BioRad, Hercules, CA) using Fast Start Universal SYBR Green (Roche Diagnostic Japan, Tokyo, Japan). PCR conditions were as follows: 50°C for 2 min, 95°C for 10 min, followed by 40 cycles of 95°C for 15 s and 60°C for 1 min. All gene-specific mRNA expression values were normalized against β-actin mRNA. The primer sequences for each gene, as well as the sizes of their products, are listed in Table 1.

**Table 1:** Oligonucleotide primers for real-time RT-PCR

Gene	Sense/anti-sense
β-Actin	5′-AGA AGA GCT ATG AGC TGC CTG ACG-3′
	5′-TAC TTG CGC TCA GGA GGA GCA ATG-3′
TH	5′-TGT GTC CGA GAG CTT CAA TG-3′
	5′-GGG CTG TCC AGT ACG TCA AT-3′
Iba1	5′-GTC CTT GAA GCG AAT GCT GG-3′
	5′-CAT TCT CAA GAT GGC AGA TC-3′
NG2	5′-TTA CCT TGG CCT TGT TGG TC-3′
	5′-GAT GAT CTG TTT GGC CTG CT-3′
PCNA	5′-TAA GTT GTC CCA GAC AAG CA-3′
	5′-GCG ATC GTC AAA GGT TTA GT-3′
GFAP	5′-CAG AAG CTC CAA GAT GAA ACC AA-3′
	5′-TCT CCT CCT CCA GCG ACT CAA C-3′
IGF-1	5′-TTG CGG GGC TGA GCT GGT GGA C-3′
	5′-GCG GTG ACG TGC CAT TTT CTG TTC-3′
HGF	5′- TCT TGG TGT CAT TGT TCC TG -3′
	5′-CCA TGG ATG CTT CAA ATA CA -3′
BDNF	5′-CGT GAT CGA GGA GCT GTT GG-3′
	5′-CTG CTT CAG TTG GCC TTT CG-3′
GM-CSFRα	5′-ACT AGT ATG TGG CTG CAG AAT TTA CTT TTC-3′
	5′-GGT ACC TCA TTT CTG GAC CGG CTT CC-3′
IL-3Rα	5′-ACT AGT ATG GTT CTT GCC AGC TCT AC-3′
	5′-GGT ACC TTA ACA TTC CAC GGT CAT AGG G-3′
Metallothionein 2	5′-CAC AGA TGG ATC CTG CTC CT-3′
	5′-GAG AAC CGG TCA GGG TTG TA-3′
Cu/Zn SOD	5′-TTC GAG CAG AAG GCA AGC GG-3′
	5′-ATC CCA ATC ACA CCA CAA GC-3′
Bcl-xL	5′-CCT ATC TTG GCT TTG GAT CC-3′
	5′-TTT CTT CTG GGG CTT CAG TC-3′
Bax	5′-TGC AGA GGA TGA TTG CTG AC-3′
	5′-GAT CAG CTC GGG CAC TTT AG-3′
TNFα	5′-CCC AGA CCC TCA CAC TCA GAT-3′
	5′-TTG TCC CTT GAA GAG AAC CTG-3′
IL-1β	5′-CAC CTT CTT TTC CTT CAT CTG T-3′
	5′-GTC GTT GCT TGT CTC TCC TTG TA-3′

TH = tyrosine hydroxylase; PCNA = proliferating cell nuclear antigen; GFAP = glial fibrillary acidic protein; IGF-1 = insulin-like growth factor-1; HGF = hepatocyte growth factor; BDNF = brain-derived neurotrophic factor; Cu/Zn SOD = copper and zinc superoxide dismutases; TNFα = tumor necrosis factor α; GM-CSFRα = granulocyte macrophage colony-stimulating factor receptor α; IL = interleukin.

### Immunoblotting

The ventral midbrain from the opposite side of the tissue used for qRT-PCR (*n* = 7) was immediately homogenized with SDS solution in 10 volumes of Laemmli's sample solution containing 3% sodium dodecyl sulfate (SDS). The lysates were electrophoresed, transferred to nitrocellulose membranes, and immunoblotted with antibodies to β-actin, tyrosine hydroxylase (TH), Iba1, NG2, and Bcl-xL (Table 2). The immunoreaction was visualized using nitro blue tetrazolium and 5-bromo-4-chloro-3-indolyl phosphate, as described previously (Tanaka et al. 1998). Immunoreactive bands were analyzed by densitometry using ImageJ 1.43u (Wayne Rasband, National Institute of Health, Bethesda, ML). The densitometry data were standardized with the internal standard β-actin.

**Table 2:** Primary antibodies used in this study

*Antigen*	*Antibody*	*Dilution*	*Source*
β-actin	Mouse monoclonal	1:1000	Sigma
TH	Sheep polyclonal	1:500	Gene tex
TH	Rabbit polyclonal	1:500	abcam
Iba1	Rabbit polyclonal	1:500	Wako
NG2	Mouse monoclonal (132.39)	1:500	Chemicon
GFAP	Rabbit polyclonal	1:500	SHIMA Laboratories
CD11b	Mouse monoclonal (MRC OX42)	1:250	Serotec
Bcl-xL	Mouse monoclonal	1:100	Transduction Laboratories
TNF-α	Rabbit polyclonal	1:500	Santa Cruz
GM-CSFRα	Rabbit polyclonal	1:500	Santa Cruz
IL-3Rα	Rabbit polyclonal	1:500	Santa Cruz
TNF-α	Rabbit polyclonal	1:100	Bioworld Technology, Inc.
IL-1β	Rabbit polyclonal	1:100	Bioworld Technology, Inc.

TH = tyrosine hydroxylase; GFAP = glial fibrillary acidic protein; TNFα = tumor necrosis factor α; GM-CSFRα = granulocyte macrophage colony-stimulating factor receptor α; IL = interleukin.

### Immunohistochemical staining

The primary antibodies listed in Table 2 were used for indirect immunofluorescence staining (Yokoyama et al. 2006). Briefly, anesthetized rats were fixed by transcardially perfusing 4% paraformaldehyde containing 2 mM MgCl_2_ for 10 min, at a flow rate of 80 mL/min. The dissected brains were immersed in 15% sucrose in PBS at 4°C overnight, rapidly frozen in dry ice powder, and sliced into 10-μm thick coronal sections at the substantia nigra level (from bregma 4.80 mm to 5.40 mm). The brain sections were incubated with the primary antibodies followed by incubation with DyLight 488, DyLight 549, and/or DyLight 649-labeled secondary antibodies (Jackson ImmunoResearch Laboratories, West Grove, PA). Hoechst 33258 (Sigma) was used for nuclear staining. The immunostained specimens were observed with a Nikon A1 confocal laser scan microscope (CLSM; Tokyo, Japan) using 20× or 60× objective lenses. The area observed was 2.0–2.3 mm lateral from the midline.

### Morphometric analysis

**Figure 6:**
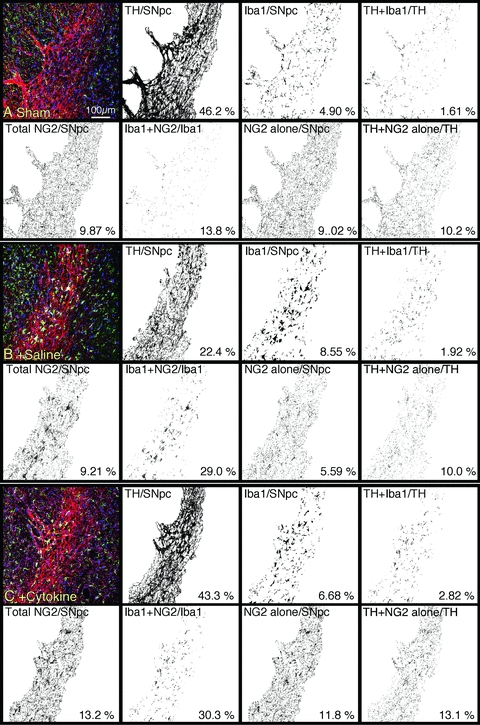
Morphometric analyses of TH^+^, Iba1^+^, and NG2^+^ cells in the SNpc of sham, saline, and cytokine-treated rats. (**A**, **B**, and **C**) Images show triple-immunostained SNpc of each group of rats (at 1 week) with antibodies to TH (red), Iba1 (green), and NG2 (pink) as merged pictures. Black and white images were drawn from the original micrographs with individual colors, after SNpc regions were picked out, based on the distribution of the TH^+^ DArgic neurons. TH/SNpc = ratio of TH^+^ region in the SNpc. Iba1/SNpc = ratio of Iba1^+^ region in the SNpc. TH+Iba1/TH = ratio of TH^+^/Iba1^+^ region in the TH^+^ region, an index of synaptic stripping by microglia. Total NG2/SNpc = ratio of NG2^+^ region in the SNpc. Iba1+NG2/Iba1 = ratio of Iba1^+^/NG2^+^ region in the Iba1^+^ region, an index of the incidence of Iba1^+^/NG2^+^ cells. NG2 alone/SNpc = ratio of NG2^+^/Iba1^−^ region in the SNpc, an index of the incidence of NG2^+^ cells that do not express Iba1. TH+NG2 alone/TH = ratio of TH^+^/NG2^+^, but not Iba1^+^ region in the TH^+^ region, an index of attached NG2^+^ cells, but not microglia, to neurons.

Brain sections processed as described above were triple-immunostained with antibodies to Iba1, TH, and NG2. To determine the area occupied by DArgic neurons, microglia, and NG2 glia, and also their overlapping area in the SNpc of the sections, micrographs were taken with the CLSM using a 20× lens. The images were processed using Adobe Photoshop CS5 Extended (Adobe Systems, San Jose, CA) and ImageJ 1.43u. First, the area where the DArgic neurons in the SNpc were distributed was demarcated as the SNpc. Then, the SNpc was further subdivided based on immunostaining for TH, Iba1, and NG2. Overlapping staining for TH and Iba1, TH and NG2, Iba1 and NG2, and Iba1-positive, but NG2-negative, were serially determined (see Fig. 6). Data from five sham or six 6-OHDA-treated rats were statistically analyzed.

### Primary cultures

Rat primary microglial cultures were prepared (Tanaka et al. 1998). Briefly, whole forebrains from neonatal rats were dissected out and dissociated into individual cells with a nylon bag with 160 μm pores. The dissociated cells were cultured as a mixed glial cell culture in 75 cm^2^ flasks with 10% foetal calf serum-supplemented Dulbecco's modified Eagle's medium. Eleven or 14 days later, microglial cells were obtained from the mixed glial culture. The purity of the microglial culture was >99%, as determined by immunocytochemical staining using antibodies to GFAP and Iba1. For immunocytochemical detection of cytokine receptors, the cells were seeded onto poly-L-lysine-coated glass coverslips placed in four-well culture plates, and then immunostained for GM-CSF and IL-3 receptors, as described above. Micrographs were taken with conventional optics using an Olympus BX-52 (Olympus, Tokyo, Japan). qRT-PCR to detect microglial mRNAs was performed in the same way as described above.

### Statistical analysis

Numerical data expressed as means ± SEM were statistically analyzed using InStat3 software (GraphPad Software, La Jolla, CA). Statistical significance was assessed with one-way analysis of variance (ANOVA) and Tukey's *post hoc* test.

## Results

### Ameliorative effects of the cytokine mixture on motor function in 6-OHDA-induced Parkinsonian rats

**Figure 1:**
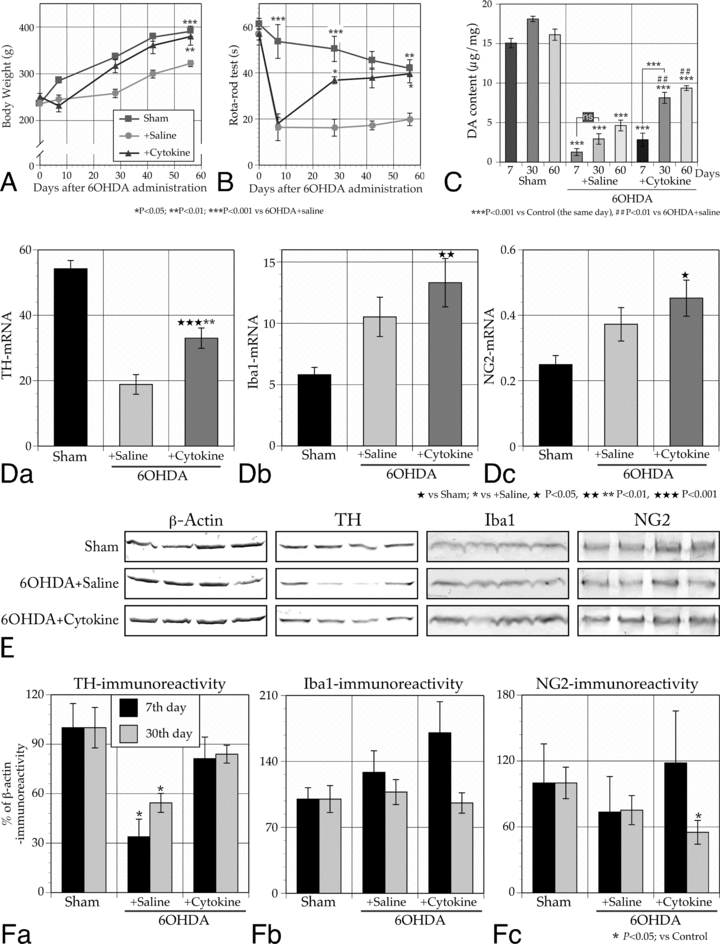
Effects of subcutaneous injection of the cytokine mixture on 6-OHDA-induced Parkinsonian rats. 6-OHDA was bilaterally administered into the striatum. +Saline or +Cytokine indicate 6-OHDA-administred rats with subcutaneous injection of saline or cytokine, respectively. Subcutaneous injection of the cytokine mixture was started 1 day after 6-OHDA administration, and repeated seven times (once per day). (**A**) Body weight change during the course of 2 months after 6-OHDA administration; *n* = 21, 21, 14, 7, and 7, at 0, 1, 4, 6, and 8 weeks, respectively, after 6-OHDA administration. (**B**) Motor function was evaluated by rota-rod test. (**C**) DA content in the striatum was determined by HPLC. The bilateral data were averaged and expressed as means ± SEM. (**D**) Levels of mRNAs encoding TH, Iba1, and NG2 in the SNpc at 1 week from seven rats, as determined by qRT-PCR. (**E**) Representative immunoblot data (*n* = 4) showing TH, Iba1, NG2, and β-actin protein level at 1 week. (**F**) Densitometric analyses of the immunoblot data expressed as means ± SEM.

The rats that received bilateral administration of 6-OHDA into the striatum did not move smoothly, and had difficulty in eating and drinking. Such motor dysfunction was apparent 6 h after 6-OHDA administration. As a result, rat body weights only minimally increased 7 days after 6-OHDA administration (Fig. 1A). After this time point, however, the 6-OHDA-lesioned rats that received the cytokine mixture injection (cytokine group) had increased body weight, nearly equivalent to the control animals’ (sham group) body weights, and the body weights of the cytokine group rats were significantly greater than the lesioned rats that received saline injection (saline group). The rota-rod test revealed marked motor dysfunction of the 6-OHDA-treated groups at 1 week (Fig. 1B). However, the cytokine group recovered motor function at 4 weeks and later. The sham group had gradually declining motor function, probably due to their increasing bodyweights. Consequently, there were no differences in motor function between the sham and cytokine groups at 8 weeks.

DA levels were measured by HPLC in the striatum. Approximately 15 μg/mg tissue weight of DA was detected in the striatum of the sham group, but less than 3 μg/mg DA was present in the 6-OHDA-treated rats (Fig. 1C). This marked decrease in DA content may underlie the motor dysfunction of the 6-OHDA-treated rats. However, DA contents of the cytokine group increased to 8 μg/mg or more, at 30 days or later.

Total RNA was prepared from the ventral midbrain containing the SNpc and then reverse transcribed into cDNA for qRT-PCR (Fig. 1D). Although 6-OHDA administration decreased the amount of mRNA encoding the rate-limiting enzyme for DA synthesis TH, the mRNA level was higher in the cytokine group than in the saline-treated group (Fig. 1Da). mRNAs for the microglia marker Iba1 and the oligodendrocyte progenitor cell marker NG2 chondroitin sulphate proteoglycan (NG2) increased in the cytokine group.

Protein samples were prepared at 7 and 30 days after 6-OHDA treatment and used for immunoblotting to estimate the amount of TH, Iba1, and NG2 at the protein level in the SNpc. Representative results from four separate samples are shown in Fig. 1E. β-actin was used as an internal standard. The protein bands from seven separate samples were analyzed by scanning densitometry. Fig. 1F shows that the TH protein decreased in the saline-treated group compared with the sham group (Fig. 1Fa). Iba1 protein tended to increase in the cytokine group at 7 days, but the level returned to the sham level at 30 days (Fig. 1Fb). NG2 protein was significantly reduced at 30 days in the cytokine group (Fig. 1Fc).

### Expression of GM-CSF and IL-3 receptors in neurons and microglia

**Figure 2:**
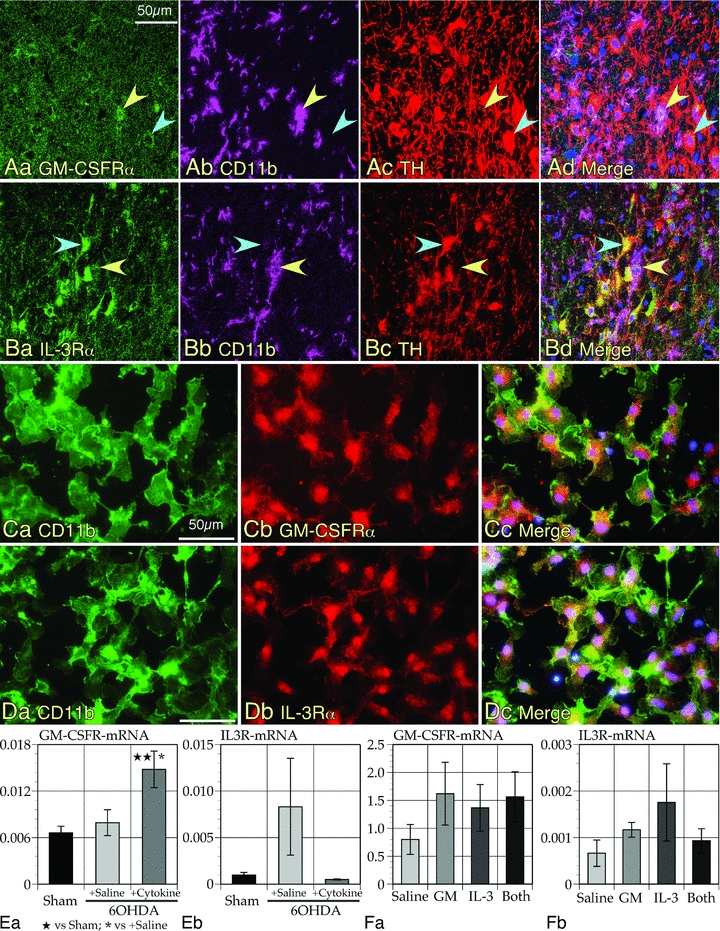
Expression of receptors for GM-CSF and IL-3 in the SNpc and primary cultured microglia. (**A**) Immunoreactivity of GM-CSFRα was localized to microglial cells (yellow arrowheads) and DArgic neurons (blue arrowheads). Microglial cells were identified with anti-CD11b antibody (OX42) and DArgic neurons with anti-TH-antibody. (**B**) Immunoreactivity of IL-3Rα was also localized to microglial cells (yellow arrowheads) and DA neurons (blue arrowheads). (**C** and **D**) Primary cultured microglial cells expressing GM-CSFRα (**C**) and IL-3Rα (**D**). (**E**) qRT-PCR revealed the presence of mRNAs encoding the receptors. The data from seven rats are expressed as means ± SEM. (**F**) The receptor mRNAs were expressed in the primary microglia culture. The data from 4 separate cultures are expressed as means ± SEM.

Antibodies to GM-CSF receptor α (GM-CSFRα) and IL-3 receptor α (IL-3Rα) were used in combination with antibodies for TH, and a marker for microglia, CD11b, to investigate localization of these receptors in the SNpc (Fig. 2). GM-CSFRα-immunoreactivity was localized both in CD11b^+^ microglia and the TH^+^ DArgic neurons (Fig. 2A). However, some microglia appeared to express the receptor more strongly than neurons. IL-3Rα immunoreactivity was also localized in both microglia and DArgic neurons (Fig. 2B), but this immunoreactivity was stronger in the neurons than in microglia. Primary cultured microglial cells expressed both receptors (Fig. 2C, D). mRNAs encoding these receptors were evaluated by qRT-PCR. Both mRNAs were detected in the ventral midbrain (Fig. 2E). Only GM-CSFRα-mRNA significantly increased in response to cytokine injection in the 6-OHDA-treated rats. This increase may imply that GM-CSFR expression is regulated in a positive-feedback manner, while IL-3R expression may likely be in a negative-feedback manner. Therefore, when simultaneously injected, GM-CSF might more effectively work than IL-3. Cultured microglial cells also expressed both mRNAs (Fig. 2F). Given the high level of expression of GM-CSFRα-mRNA in the cultured microglia, it is likely that the main source of GM-CSFRα-mRNA in the ventral midbrain is microglia. On the other hand, DArgic neurons may be the main source of IL-3Rα-mRNA.

### Increased expression of Bcl-xL in DArgic neurons in the SNpc of the cytokine-injected rats

**Figure 3:**
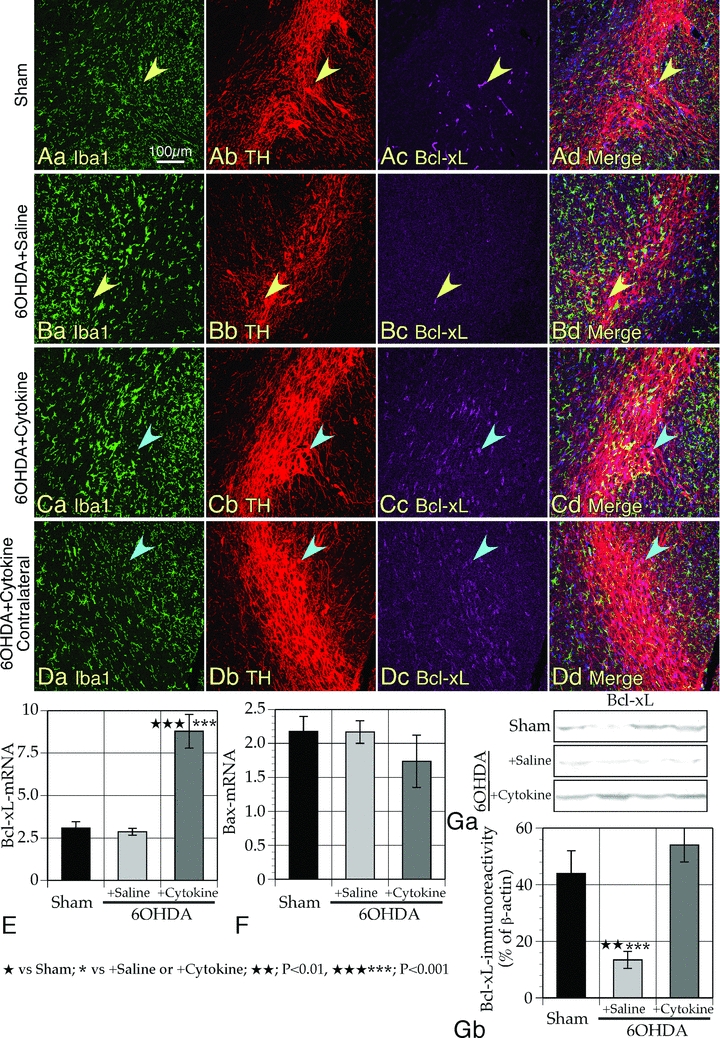
Antiapoptotic factor Bcl-xL expression in the SNpc. (**A–D**) Representative immunohistochemical data showing expression of Bcl-xL protein in the SNpc of sham, saline, and cytokine group at 1 week after 6-OHDA administrations. Localization of Bcl-xL immunoreactivity was only found in the cytokine-injected rat SNpc. The upregulated Bcl-xL expression was also found in the contralateral SNpc, where microglia display the resting phenotype. (**E**) Bcl-xL mRNA level was elevated in the cytokine group at 1 week, as revealed by qRT-PCR (*n* = 7). (**F**) Bax-mRNA did not show marked differences among the three groups. (**G**a) Bcl-xL protein was strongly detected in the cytokine group by immunoblotting, while its expression was suppressed in the saline group. Representative data from four rats of each group are shown. (**G**b) Densitometric analysis showed a significant drop in Bcl-xL protein expression in the saline group. Data from seven rats of each group are expressed as means ± SEM.

Both GM-CSF and IL-3 have been reported to increase the expression of antiapoptotic factors belonging to the Bcl-2 family in isolated neurons (Wen et al. 1998; Huang et al. 2007; Schabitz et al. 2008). Immunohistochemical staining with antibodies to Iba1, TH, and Bcl-xL revealed that Bcl-xL immunoreactivity was localized to capillary-like structures (yellow arrowheads) in and around the SNpc in a sham rat (Fig. 3A). Bcl-xL-immunoreactivity was similarly localized in a saline-injected Parkinsonian rat, although the immunoreactivity was markedly suppressed (Fig. 3B). By contrast, strong Bcl-xL-immunoreactivity was localized to DArgic neurons of a cytokine-injected rat (Fig. 3C, blue arrowheads). Note that the activated morphology of microglia was found in the SNpc, only in the ipsilateral side of the 6-OHDA-treated rats. Furthermore, immunoreactivity at a similar level was observed in DArgic neurons in the contralateral SNpc of the cytokine-injected rat, where microglia display resting ramified morphology (Fig. 3D). qRT-PCR showed a significant increase of Bcl-xL-mRNA in the cytokine group (Fig. 3E), and the proapoptotic factor Bax-mRNA did not significantly change among the three groups (Fig. 3F). In comparison with the mRNA data, Bcl-xL protein was not increased in the cytokine group compared with the sham group. However, the Bcl-xL protein was markedly decreased in the saline group (Fig. 3G). These data suggest that 6-OHDA administration accelerates degradation of Bcl-xL protein and that the cytokine injection increased the transcription of Bcl-xL mRNA in DArgic neurons.

### Phenotypic changes of microglia in response to the cytokines

**Figure 4:**
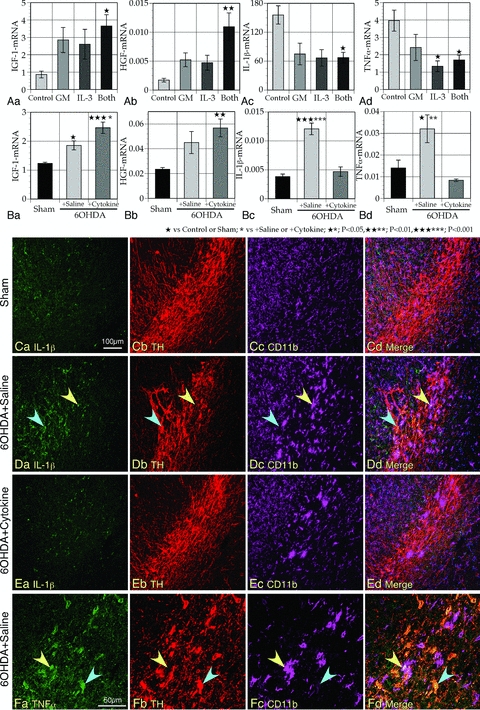
Effects of cytokines on microglial cells *in vitro* and *in vivo*. (**A**) qRT-PCR revealed that cultured microglial cells have increased mRNAs encoding IGF-1 (**A**a) and HGF (**A**b), while there is decreased mRNAs encoding IL-1β (**A**c) and TNFα (**A**d), when they were incubated with the cytokine mixture. The *in vitro* data were obtained from four separate cultures and expressed as means ± SEM. (**B**) mRNAs encoding IGF-1 (**B**a) and HGF (**B**b) also increased in tissue containing SNpc in response to the cytokine injection. By contrast, mRNAs for IL-1β (**B**c) and TNFα (**B**d) increased only in the saline group. *In vivo* qRT-PCR was from seven rats and expressed as means ± SEM. (**C–E**) IL-1β immunoreactivity in the SNpc of sham (**C**), saline (**D**), and cytokine (**E**) group at 1 week after 6-OHDA administrations. Rather strong IL-1β immunoreactivity was localized in neurons (blue arrowheads) and microglia (yellow arrowheads) in SNpc of saline-injected rats. (**F**) TNFα immunoreactivity was found in microglial cells (yellow arrowheads) and in neurons (blue arrowheads) in SNpc of saline-injected rats.

It has been shown previously that primary cultured rat microglial cells change their morphology in response to GM-CSF and IL-3 (Fujita et al. 1996). To determine whether primary microglial cells change their functional phenotypes in response to the cytokines, total RNA was prepared from microglial cells cultured in the presence or absence of the cytokines, and mRNA levels encoding insulin-like growth factor-1 (IGF-1), hepatocyte growth factor (HGF), IL-1β, and TNFα were determined (Fig. 4A). Both IGF-1 and HGF ameliorate 6-OHDA-induced Parkinsonism (Clarkson et al. 2001; Koike et al. 2006; Ebert et al. 2008). mRNAs encoding the neuroprotective factors IGF-1 and HGF increased, and mRNAs for the detrimental proinflammatory cytokines decreased in the presence of the both cytokines. Thus, this result indicates that GM-CSF and IL-3 strengthened the neuroprotective nature of cultured microglia. Similar results were obtained in *in vivo* experiments (Fig. 4B). IGF-1 and HGF-mRNAs increased more significantly in the ventral midbrain of the cytokine group than in the ventral midbrain of the saline group. IL-1β and TNFα mRNAs markedly increased in the saline group, but the levels in the cytokine groups returned to the sham level. Immunohistochemical staining using anti-IL-1β and TNFα antibodies showed the positive immunostaining of the proinflammatory cytokines in DArgic neurons (blue arrowheads) and microglia (yellow ones) (Fig. 4C–F). In spite of such functional differences, microglial cells in the SNpc displayed amoeboid morphology in both the 6-OHDA-treated groups.

### Contact between neurons and glia

**Figure 5:**
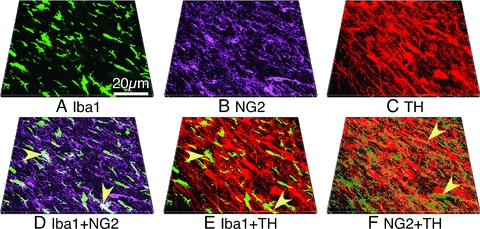
Reaction of cells expressing Iba1 and NG2 in the SNpc of a cytokine-injected 6-OHDA-administred rat, as shown in a 3D-reconstructed immunohistochemical picture. Seventeen 0.25-μm thick optical sections were reconstructed. (**A**, **B**, and **C**) Images show Iba1^+^ (green), NG2^+^ (pink), TH^+^ (red) cells, respectively. (**D**) Iba1^+^/NG2^+^ cells, denoted with arrowheads, were present, displaying microglia-like morphology. (**E**) Iba1^+^ microglial cells often are closely attached to TH^+^ DA neurons (arrowheads), appearing as a merge of green and red colors to produce the yellow color. (**F**) Attachment of NG2^+^ cells to TH^+^ DA neurons. Color of NG2^+^ cells was changed to green, and then merged with red colors. A closely attaching region between DA neurons and NG2^+^ cells is displayed as an orange color (arrowheads).

Detailed morphological observation using 3D-constructed images taken by CLSM revealed the intimate contacts between neurons and glial cells and the presence of Iba1^+^/NG2^+^ cells (Fig. 5). The brain section in Figure 5 was from a cytokine-injected rat that was immunostained with antibodies to Iba1, NG2, and TH. The merged image of Iba1 and NG2 immunoreactivities (Fig. 5D) shows the presence of Iba1^+^/NG2^+^ cells, which have been described as a neuroprotective cell type (Kitamura et al. 2010). Activated microglial cells have long been described to intimately attach to damaged neurons and remove synaptic inputs. This phenomenon is called “synaptic stripping” and is supposed to be neuroprotective (Cullheim and Thams 2007; Trapp et al. 2007). The presence of synaptic stripping by immunofluorescence would be evident when the green fluorescence representative of Iba1-immunoreactivity is merged with the red fluorescence of TH-immunoreactivity, thus producing yellow color. Indeed, the merged yellow color is evident in the region where microglia and DArgic neurons intimately attach in Fig. 5E. In addition, NG2 glia also appeared to closely attach to DArgic neurons. This is seen when green immunofluorescence of NG2 is merged with the red immunofluorescence of DArgic neurons; the contact regions of NG2 glia and DArgic neurons appear as orange regions (Fig. 5F). The attachment of NG2 glia to DArgic neurons appeared more frequently than that of microglia.

### Morphometric analyses of cell densities and interactions between glial cells and DArgic neurons

For the statistical evaluation of the cell types in the different treatments, detailed morphometric analyses were conducted using the CLSM images of Iba1, NG2, and TH immunofluorescence. Because immunoblotting and qRT-PCR was done on dissected ventral midbrain that not only contained the SNpc, but also other regions, it was necessary to employ immunohistochemical technique to analyze specific reactions of cells selectively in the SNpc. The region containing TH-immunoreactivity was defined as the SNpc region, and the area was determined using ImageJ 1.43u software. Similarly, areas containing TH, Iba1, and NG2 immunoreactivities were also independently measured. Furthermore, the following overlapping stained areas were also measured: TH/Iba1-double-positive areas (indicative of synaptic stripping by microglia), Iba1^+^/NG2^+^-double-positive areas (indicative of NG2^+^ microglia), Iba1^−^/NG2^+^ areas (indicative of NG2 glia that are not microglia), and TH^+^/Iba1^−^/NG2^+^ areas (indicative of attachment of NG2 glia to DArgic neurons). These areas were further divided by areas positive for SNpc, TH, or Iba1. Figures 6 A–C show examples of processed pictures from these morphometric analyses.

**Figure 7:**
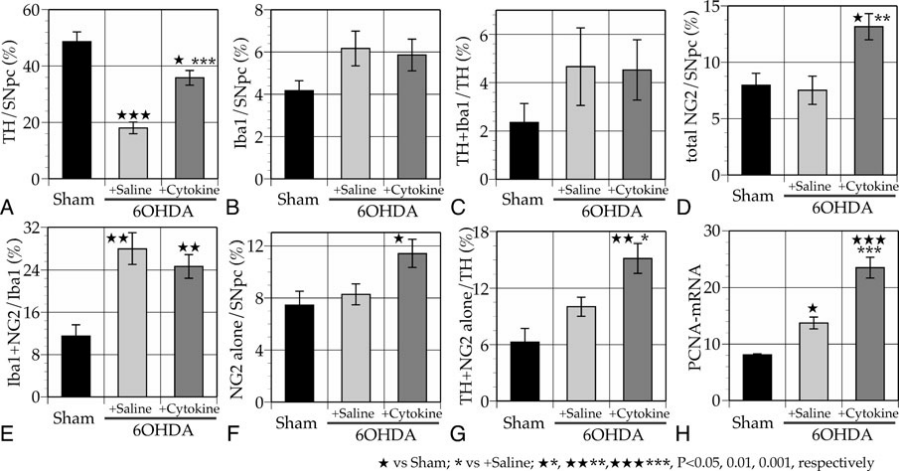
Based on the processed micrographs as shown in Figure 6, the morphometrical data from the sham group (*n* = 5) or saline (*n* = 6) and cytokine groups (*n* = 6) were statistically analyzed and expressed as means ± SEM. PCNA-mRNA level are also shown. (**A**) TH-immunoreactivity detected in the cytokine group was intermediate among the three groups. (**B**) In the SNpc, an increase of the Iba1^+^ region, which is an index of microglial activation, was not significant in the Parkinsonian rats. (**C**) TH^+^/Iba1^+^ region, which is an index of synaptic stripping by microglia, was also not significantly increased. (**D**) Total NG2^+^ region was increased in the cytokine group. (**E**) Iba1^+^/NG2^+^ region, which is an index of NG2^+^ microglia, was increased in both the saline and cytokine groups. (**F**) NG2^+^ cells that are not microglia increased in the cytokine group. (**G**) Cytokine injection increased TH^+^/NG2^+^ alone region occupied total TH^+^ region, which is an index of NG2 cell attachment to DArgic neurons. (**H**) PCNA-mRNA level was elevated in 6-OHDA-treated rats, but the elevation was more significant in the cytokine group.

Summaries of the results obtained from the sham group (five rats) and the saline and cytokine groups (six rats) are shown in Figures 7A–G. TH/SNpc data are indicative of the number of surviving DArgic neurons in the SNpc (Fig. 7A), which was comparable to the immunoblot and RT-PCR data shown in Figure 1D–F. Cytokine injection significantly prevented DArgic neuronal loss. Iba1/SNpc is indicative of microglial activation (Fig. 7B). However, the Iba1^+^ area in the SNpc was not markedly expanded, even in the 6-OHDA-treated rats, in spite of the presence of activated microglia. Furthermore, there was no difference in the Iba1^+^ area between the saline and cytokine groups. TH+Iba1/TH is indicative of synaptic stripping (Fig. 7C), but there were no significant differences among the three groups. Total NG2/SNpc is indicative of the degree of activation and/or proliferation of NG2^+^ cells, which includes NG2^+^ microglia (Fig. 7D). This index significantly increased only in the cytokine group. The level of Iba1+NG2/Iba1 significantly increased in the 6-OHDA-treated rats as described elsewhere (Kitamura et al. 2010), both in the saline and cytokine-treated groups (Fig. 7E), suggesting that NG2^+^ microglia do not contribute to the cytokine-induced DArgic neuronal survival. The NG2 alone/SNpc index increased only in the cytokine group (Fig. 7F), indicating that NG2 glia increased in number only in the cytokine group. TH+NG2 alone/TH index also increased only in the cytokine group (Fig. 7G). These morphometric data suggest that the increase of NG2 glial cell number and their attachment to DArgic neurons may underlie the neuroprotective effects of the cytokine mixture. mRNA encoding proliferating cell nuclear antigen (PCNA, a marker for proliferating cells) was markedly increased in the cytokine group, which may be indicative of increased NG2 glial cell numbers rather than microglia.

### Astrocytes and astrocyte-related factors in the SNpc

**Figure 8:**
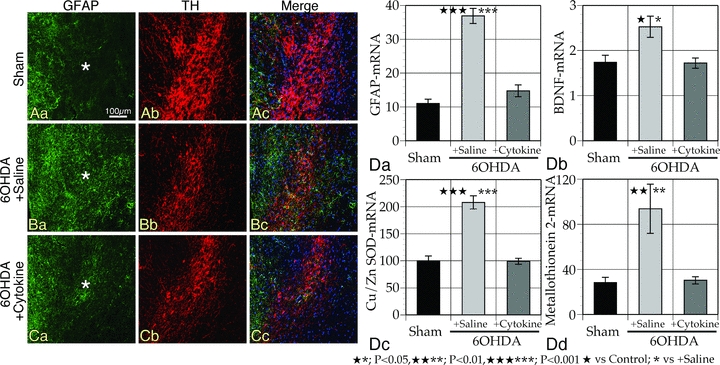
Reaction of astrocytes in the SNpc. (**A**) GFAP-immunoreactivity was weak in the SNpc (denoted with an asterisk) of sham-treated rats. (**B**) Strong GFAP-immunoreactivity was observed in saline-injected rats. (**C**) Moderate GFAP-immunoreactivity was observed in cytokine-injected rats. (**D**) mRNA levels encoding factors related to astrocytes as determined by real-time RT-PCR. GFAP-mRNA increased in the saline-injected group (**D**a). BDNF- (**D**b), Cu/Zn SOD- (**D**c), and metallothionein 2 (**D**d) mRNAs also increased in the saline-injected group. The data were obtained from seven rats.

Double-immunohistochemical staining using antibodies to GFAP and TH was done to evaluate astrocytes in the SNpc (Fig. 8). In the SNpc of the sham rats, GFAP immunoreactivity was scarcely distributed (Fig. 8A). In contrast, GFAP immunoreactivity was increased in the saline-injected rats (Fig. 8B). However, GFAP immunoreactivity was noticeably reduced in cytokine-injected rats (Fig. 8C). In agreement with these morphologic observations, GFAP-mRNA was also increased only in the saline group. mRNAs encoding BDNF, Cu/Zn SOD, and metallothionein 2, which could most likely be from astrocytes, were increased only in the saline group.

## Discussion

This study demonstrated that subcutaneous administration of a cytokine mixture of GM-CSF and IL-3 exhibited marked neuroprotective effects against 6-OHDA-induced Parkinsonism in rats. It is of clinical relevance that the cytokine administration was started one day after the 6-OHDA-treatment. The dose of the cytokines was 10 μg/kg bodyweight, which is comparable to the dose of GM-CSF or IL-3 typically used for human cases to stimulate the bone marrow (Hocker et al. 1993; Bastion et al. 1995). Based on these facts, the cytokine mixture used in the present study may be clinically applicable for the treatment of PD. Furthermore, given the marked effects of this cytokine mixture in a model of PD, it can also be employed as a pharmacological tool to determine therapeutic targets to prevent PD-associated neuronal death.

Previously, it was shown that GM-CSF upregulates the expression of antiapoptotic factors belonging to the Bcl family in neurons expressing GM-CSFR (Huang et al. 2007; Schabitz et al. 2008), which resulted in the prevention of neuronal cell death. IL-3 was also shown to suppress neurodegeneration through increased Bcl-xL expression (Wen et al. 1998). In this study, subcutaneous injection of the cytokine mixture induced DArgic neurons in 6-OHDA-lesioned brains to upregulate Bcl-xL expression. This effect was independent of activated microglia, because DArgic neurons in the contralateral SNpc also upregulated expression of Bcl-xL, as shown in Figure 3. This likely contributed to suppressed DArgic neurodegeneration. The surviving neurons with elevated Bcl-xL expression then would affect the actions of glial cells within their vicinity. Neurons fated to die may activate microglial cells to accelerate neuronal degeneration, while surviving neurons may activate neuroprotective attributes of glial cells (Streit et al. 1999; Cullheim and Thams 2007).

Regardless of the cause of brain injury, microglial cells respond to even minor pathologic events in the brain, evident by morphologic changes such as enlargement of their cell bodies and shortening of their ramified processes (Kreutzberg 1996; Streit et al. 1999). 6-OHDA-treatment caused microglial activation with these types of morphologic changes. However, it is notable that the activated morphology was observed regardless of the survival of DA neurons, with or without the cytokine mixture injection. Interestingly, many studies have described the harmful effects of activated microglia on neurons (Mosley et al. 2006; Long–Smith et al. 2009; Tansey and Goldberg 2010); however, our results suggest that this may not always be the case.

There are some controversies regarding the activation of microglia (Liberatore et al. 1999; Henry et al. 2009; Marinova–Mutafchieva et al. 2009). Is their activation the cause or the result of DArgic neurodegeneration? Because DArgic neurodegeneration induced by 6-OHDA is a rather chronic process (Henry et al. 2009; Marinova–Mutafchieva et al. 2009), it is conceivable that microglial activation may influence the fate of DArgic neurons even if the DArgic neurodegeneration precedes microglial activation. In fact, proinflammatory cytokines, such as IL-1β or TNFα, and reactive oxygen species, such as NO or superoxide, which are produced by microglia, have been implicated in the pathogenesis of PD (McGeer and McGeer 2008; Long–Smith et al. 2009; Yacoubian and Standaert 2009; Tansey and Goldberg 2010).

The ameliorative effects of the cytokine mixture may be related to the functional changes of the activated microglia. The cytokine injection decreased the expression of IL-1β or TNFα in the SNpc of 6-OHDA-treated rats and it simultaneously increased expression of IGF-1 and HGF. IGF-1 (Guan et al. 2000; Ebert et al. 2008;) and HGF (Koike et al. 2006) have been shown for its ameliorative effects of 6-OHDA-induced rat Parkinsonism. Addition of GM-CSF and IL-3 to primary microglial cell cultures induced similar expression spectra of the proinflammatory cytokines and the neuroprotective factors. Thus, the action of the cytokine mixture to alter the microglial phenotype from a neurotoxic phenotype to a neuroprotective one, could at least partly explain the amelioration of 6-OHDA-induced Parkinsonism by the cytokine mixture. Damaged DArgic neurons can cause microglial activation through soluble factors, such as μ calpain (Levesque et al. 2010), and other insoluble factors on the plasma membrane (Sudo et al. 1998). Microglia activated by signals from damaged neurons may produce harmful factors that further contribute to neurodegeneration, or by phagocytizing the dying neurons. However, when the neuronal damage is not severe enough to induce neuronal death, microglia may become neuroprotective and promote neuronal survival by releasing various neuroprotective factors. This duality of function by microglia has long been proposed (Kreutzberg 1996; Streit et al. 1999; Cullheim and Thams 2007), and agents that change the microglial phenotype from destructive to protective have been sought for a long time as treatments for neurological disorders. This cytokine mixture may have this microglial phenotype-changing activity. The beneficial effect of this cytokine mixture may also be related to its ability to increase the expression of Bcl-xL in neurons. This effect may promote the survival of damaged neurons, activate the neuroprotective actions of surrounding microglia, and further bolster neuronal survival.

Expression of NG2 by microglia may be another hallmark of their activation (Yokoyama et al. 2006; Kitamura et al. 2010; Zhu et al. 2010). Although NG2^+^ microglia have been reported to express a neuroprotective factor, GDNF (Kitamura et al. 2010), it appears that in the present scenario this neuroprotective factor did not contribute to neuronal survival in the 6-OHDA-induced Parkinsonism model. This is because NG2^+^ microglia were present following 6-OHDA treatment without and with cytokine treatment.

6-OHDA-induced neurotoxicity has been attributed to oxidative stress (Glinka et al. 1997). Astrocytes have strong antioxidant properties (Tanaka et al. 1999; Miyazaki et al. 2011), and activated astrocytes are known to prevent DArgic neurodegeneration (Asanuma et al. 2010; Choudhury et al. 2011). Activated astrocytes were also evident in this study and the expression of mRNAs encoding Cu/Zn SOD and metallothionein 2, both of which play critical roles in suppressing oxidative stress, were upregulated in parallel with increased GFAP expression in the SNpc of the saline group. However, the activation of astrocytes and the upregulation of antioxidant factors did not lead to improved survival of neurons. Furthermore, when neurodegeneration was suppressed with the cytokine mixture, both astrocytic activation and the expression of antioxidative factors were also suppressed, suggesting that astrocytes and the antioxidative factors do not contribute to DArgic neuronal survival in the presence of the cytokines.

On the other hand, NG2 glia may contribute to the survival of DArgic neurons. NG2 glia are abundantly distributed throughout the brain and the spinal cord, representing 5–15% of nonneuronal cells (Staugaitis and Trapp 2009; Trotter et al. 2010). Some of these cells are also oligodendrocyte progenitor cells. However, it is not clear so far how these cells respond to neural injury in PD. As shown in the present study, NG2 glia appeared to increase in number and to attach intimately to damaged DArgic neurons in the SNpc in the cytokine group. Elevation of PCNA-mRNA may be related to the proliferation of NG2 glia. In contrast to astrocytes, the increase in the occupying area by NG2 glia and their attachment to DArgic neurons were prominent in the cytokine group, and therefore, it is possible that NG2 glia elicit neuroprotective effects under the influence of the cytokine mixture. However, NG2 glia did not express receptors for GM-CSF, and IL-3. NG2 glia may respond to IGF-1 and HGF released by microglia. IGF-1 has been shown to be crucial for the survival of NG2 glial cells (Sundberg et al. 2010). NG2 glia express c-Met/HGF receptor, and HGF promotes NG2 glial proliferation (Ohya et al. 2007). In the present study, the cytokine mixture was found to upregulate expressions of IGF-1 and HGF in cultured microglia and in microglia in the ventral midbrain. Therefore, the cytokine mixture may stimulate NG2 glial survival and proliferation through IGF-1 and HGF, which is released by microglial cells in the SNpc of the cytokine-treated rats.

**Figure 9:**
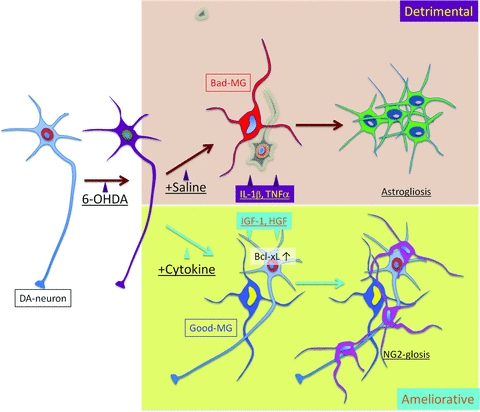
A summarized scheme based on the present data. 6-OHDA-induced DArgic neurodegeneration accompanies microglial activation. Without the cytokine mixture injection, activated microglia (MG) release the proinflammatory cytokine IL-1β and TNFα, causing further damage to DA neurons, and results in the formation of astrogliosis. Therefore, the activated microglia in this case can be considered as “Bad MG.” When the cytokine mixture is injected, increased Bcl-xL expression suppresses DA neurodegeneration, and activated MG does not cause appreciable release of detrimental proinflammatory cytokines, but neuroprotectants, such as IGF-1 and HGF. Therefore, they can be considered as “Good MG.” IGF-1 and HGF from activated MG can cause activation and proliferation of NG2 glia.

In conclusion, this study demonstrated the neuroprotective effects of a cytokine mixture containing GM-CSF and IL-3. A summary of our findings is shown in Figure 9. We propose that 6-OHDA administration into the striatum causes DArgic neurodegeneration in the SNpc and accompanying microglial activation (Fig. 9). The activated microglia produce proinflammatory cytokines that cause further chronic neurodegeneration. This neurodegeneration may also cause further activation of microglia, which in this scenario is not neuroprotective. Thus, a vicious cycle of neuronal degeneration occurs (Levesque et al. 2010). On the other hand, when the cytokine mixture is injected, DArgic neurons increase Bcl-xL expression, and thus, these neurons avoid degeneration in the face of 6-OHDA toxicity. In this scenario, the microglia become activated and display an activated morphology, similar to that in the saline group, but in this case they suppress proinflammatory cytokine expression. The microglia in the cytokine mixture-treated group have enhanced expression of the neuroprotective factors IGF-1 and HGF. IGF-1 and HGF enhances not only the viability of neurons but also the survival and production of NG2 glia, which can contribute to neuronal survival. Therefore, it is proposed that this cytokine mixture has neuroprotective properties and could help in the treatment of PD.
